# Transient acquisition process of grapheme-color synesthesia on novel graphemes

**DOI:** 10.3389/fpsyg.2026.1821750

**Published:** 2026-08-11

**Authors:** Asaka Ishikawa, Seiichiro Katsura, Eiko Matsuda

**Affiliations:** Department of System Design Engineering, Keio University, Yokohama, Japan

**Keywords:** eye tracking, gaze entropy, grapheme-color synesthesia, longitudinal learning, temporal consistency

## Abstract

Synesthesia is a cognitive trait in which stimulation in one sensory modality elicits an additional experience in another modality. Although both genetic and environmental factors have been proposed to contribute to the development of synesthesia, longitudinal studies focusing on the acquisition phase remain scarce. In the present longitudinal eye-tracking study, we examined whether gaze entropy reflects learning-related changes during the acquisition of a novel grapheme system (Thai numerals). Self-reported grapheme-color synesthetes with behavioral consistency evidence completed three laboratory sessions combined with at-home training while selecting colors for Thai and Arabic numerals. To quantify the degree of competition among candidate colors, Shannon entropy was calculated from fixation distributions. A linear mixed-effects model revealed a significant interaction between session and stimulus language, indicating that entropy decreased across sessions for Thai numerals, whereas entropy for well-learned Arabic numerals remained stable. This reduction suggests that gaze progressively converged toward fewer color candidates during learning.

## 1 Introduction

Synesthesia is a phenomenon in which stimulation in one sensory modality simultaneously elicits a percept in another modality. For example, some synesthetes experience colors when viewing graphemes or perceive shapes when hearing sounds, and a wide variety of inducer–concurrent pairings across sensory modalities has been reported (Baron-Cohen, 1996; Ward, 2013; Mills et al., 2003; Chiou and Rich, 2014). Hallmark characteristics of synesthesia include (1) long-term consistency of the inducer–concurrent associations, (2) idiosyncrasy at the individual level, (3) automaticity, and (4) rarity in the general population (Ward, 2013; Deroy and Spence, 2013; Chiou et al., 2013).

A range of studies suggests that while genetic and personality factors influence the propensity to have synesthesia, the specific mapping between inducers and concurrents is shaped by environmental factors (Galton, 1883; Barnett and Newell, 2008; Asher et al., 2009; Tilot et al., 2018; Rouw and Scholte, 2016; Chun and Hupé, 2016; Ward, 2008, 2019). Twin studies show that concordance for the presence of synesthesia is significantly higher within twin pairs; yet, grapheme–color correspondences remain idiosyncratic, even between monozygotic twins (Bosley and Eagleman, 2015). Family studies indicate that 42% of synesthetes report a first-degree relative with synesthesia, and that different synesthesia types can occur within the same family (Barnett and Newell, 2008). In studies

of grapheme–color synesthesia among children aged 6–7 years, 1.3% of child participants were identified as synesthetes; because this prevalence is comparable to that in adults, it suggests that the presence of synesthesia is already determined by ages 6–7 (Simner et al., 2009; Simner, 2013). At the same time, although child synesthetes exhibit greater temporal consistency than non-synesthetic peers, their consistency is still lower than that of adult synesthetes, implying that specific associations are formed and stabilized through learning and development. Taken together, these findings suggest that while the possession of synesthesia is innately determined, particular mappings progress from a dynamic and unstable phase to a static and stable state.

Experimental studies have suggested that inducer–concurrent correspondences are unstable during the transitional phases of synesthetic development, yet research that visualizes these transitional states remains scarce. One reason is that synesthesia has long been quantified primarily using temporal-consistency tests based on self-reports (Baron-Cohen, 1996; Ward, 2013). Historically, because synesthesia relies on introspective reports, its behavioral validity was questioned. However, the existence of individuals who can reproduce highly precise grapheme–color correspondences across repeated sessions over time established synesthesia as a legitimate object of scientific inquiry (Baron-Cohen, 1996). However, while temporal consistency is sufficient to measure the post-convergence state of synesthesia, it is ill-suited to detect the unstable dynamics during the formation process of it.

To address this gap, we introduce gaze-based process measures. In research on preference and decision-making, gaze patterns reflect the pre-decisional dynamics of competition and convergence (Shimojo et al., 2003), and gaze is integrated into the computation and comparison of value (Krajbich et al., 2010). Accordingly, gaze provides a promising index of competitive/convergent dynamics that precede introspective reports (Orquin and Loose, 2013). More broadly, eye-tracking measures have been widely used to quantify perceptual and attentional processes related to color perception, visual discrimination, and preference formation. Metrics such as fixation distributions, scanpaths, and gaze allocation provide objective indices of how perceptual representations evolve over time (Bortolotti et al., 2025b). In particular, the Shannon entropy of gaze distributions has been used as a summary measure of uncertainty or degree of competition – whether fixations are dispersed across many options or concentrated on a few (Shic et al., 2008; Krejtz et al., 2015; Di Stasi et al., 2016). We apply gaze entropy to the formation of grapheme–color correspondences and, together with conventional temporal consistency based on self-report, longitudinally test the hypothesis that competition during the transitional period decreases with learning.

Specifically, we longitudinally track synesthetes as they learn novel graphemes (Thai numerals) and examine the dynamics of mapping formation by combining (a) gaze entropy with (b) temporal consistency based on self-reports. At each learning stage, digits (Arabic/Thai) are printed in 24 basic colors, and biases in fixation toward each color region are used to quantify the degree of convergence in the emerging mappings. This approach enables quantification of the dynamic trajectory toward a stable state, proposes an alternative metric to temporal consistency for measuring grapheme–color correspondences, and offers a perspective that reconceptualizes synesthesia as a dynamic correspondence.

## 2 Materials and methods

### 2.1 Participants

We collected data from five participants recruited via personal contacts and posters displayed on campus (one male, four female; mean age = 26.0 years, ± 11.3 SD; one age unreported). Inclusion was based on self-reported grapheme–color synesthesia; we did not administer a formal consistency-based diagnostic (e.g., Synesthesia Battery). As a within-task validity check, participants showed high temporal consistency for Arabic numerals across sessions (see Section 3). None of the participants had previously learned Thai numerals.

Because grapheme–color synesthesia is relatively rare and recruitment is challenging, one of the authors (EM) was retained in the analysis as Participant S-3; potential expectancy effects are considered as a limitation. One participant (S-1) withdrew during the experiment; analyses, therefore, include four participants.

The study was reviewed and approved by the Research Ethics Committee of the Faculty of Science and Technology and the Graduate School of Science and Technology at Keio University (Project Number: 2024-109). All participants provided written informed consent in accordance with the principles outlined in the Declaration of Helsinki.

### 2.2 Stimuli

The stimuli consisted of Thai and Arabic numerals from zero to nine. The correspondence between Thai and Arabic numerals is shown in Table 1. In addition, a set of 24 colors was used as color stimuli. These colors comprised 12 basic hues and an additional 12 colors with reduced overall luminance. The specific RGB values of all colors are provided in Supplementary Table S1.

**Table 1:** Thai numerals used in the study (from zero to nine).

Language	Numerals									
Arabic	0	1	2	3	4	5	6	7	8	9
Thai	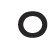	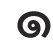	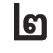	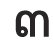	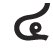	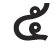	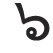	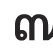	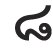	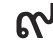

### 2.3 Procedure

**Figure 1:**
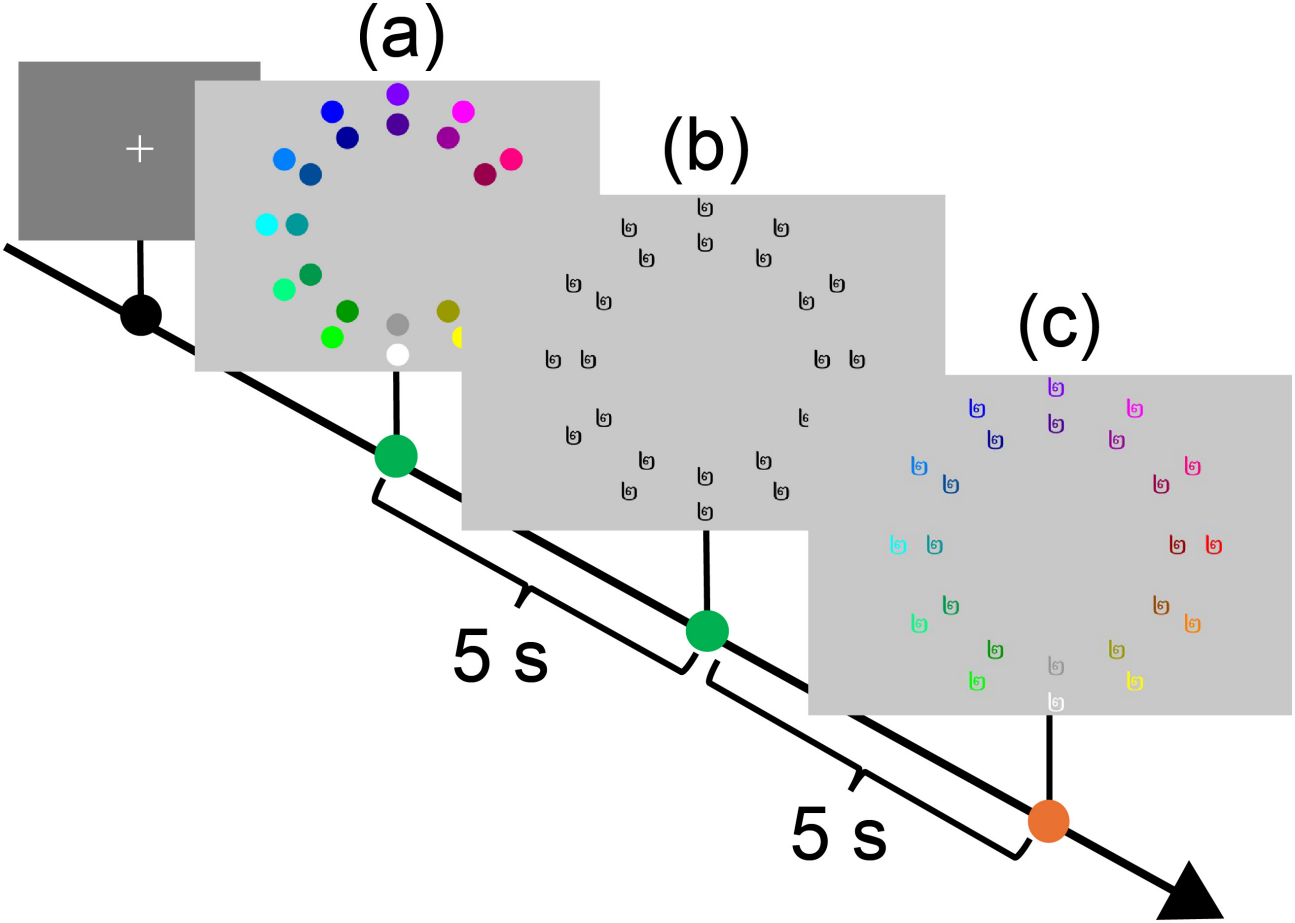
Procedure of the task. After a calibration procedure, **(a)** a circular array of colored dots forming a hue circle was shown for 5 s (free viewing), followed by **(b)** a display of black Thai numerals for 5 s (free viewing). Next, **(c)** a display containing Thai numerals in multiple colors was presented, in which participants were instructed to indicate the synesthetic color they perceived.

The experiment was designed to combine laboratory sessions with at-home learning tasks. The experimental schedule was as follows:

Day 1: First laboratory session (Figure 1).

Days 2–8: First at-home learning period (Supplementary Figure S1a).

Day 9: Second laboratory session (Figure 1).

Days 10–19: Second at-home learning period (Supplementary Figure S1b).

Day 20: Third laboratory session (Figure 1).

#### 2.3.1 Laboratory session

During the laboratory sessions, participants were presented with visual stimuli in the sequence illustrated in Figure 1. After completing the eye-tracker calibration procedure,

(a) A set of 24 colors arranged on a color wheel was presented for 5 s (Figure 1a).
(b) A Thai numeral (or an Arabic numeral) printed in black was presented for 5 s (Figure 1b).
(c) The same Thai numeral (or Arabic numeral) shown in (b) was then displayed in each of the 24 colors and arranged on the color wheel (Figure 1c).

Participants were instructed in advance to click on the numeral displayed in the color that they felt best matched the numeral during step (c). During the selection process, participants were encouraged to verbalize their thoughts, and their utterances were audio-recorded. After a participant selected one of the color options, the procedure returned to step (a), and the next numeral was presented.

The numerals used as stimuli (Thai or Arabic) consisted of the ten numerals from zero to nine, which were presented sequentially. After completing all Thai numerals, the same procedure was repeated for Arabic numerals. Throughout the entire procedure, participants' eye movements were continuously recorded using an eye tracker.

#### 2.3.2 At-home learning

The at-home learning phase was divided into two periods: an initial phase and a subsequent phase. The initial phase was conducted for 7 days between the first and second laboratory sessions, and the subsequent phase was conducted for 10 days between the second and third laboratory sessions. The learning materials used for the at-home training are shown in Supplementary Figures S1, S2 (S1: initial phase; S2: subsequent phase).

During the initial phase, the goal was to learn the correspondence between the shapes and meanings of the numerals. Participants engaged in numeral tracing exercises and training tasks that associated numeral shapes with their phonological representations. During the subsequent phase, the goal was to deepen participants' understanding of the numerical meanings and to enable practical use of the numerals. Accordingly, participants practiced basic arithmetic operations, including addition and subtraction.

To allow the experimenter to monitor daily task completion, participants were required to complete an online review task after finishing their daily worksheet-based learning. In this review task, numbers were presented auditorily, and participants were asked to select the corresponding numeral.

### 2.4 Experimental environment

Eye-tracking measurements were conducted in a quiet laboratory environment. The stimulus display was presented on a FlexScan EV2495-WT monitor (EIZO Corporation, Hakusan, Ishikawa, Japan), and the height of the display was adjusted using a height-adjustable desk so that the center of the screen was aligned with the participant's eye level. Eye movements were recorded using a Tobii Pro Fusion eye tracker (Tobii Technology, Tobii AB, Stockholm, Sweden) with a sampling rate of 60 Hz.

### 2.5 Learning acquisition assessment

Learning outcomes were assessed using a listening test and arithmetic tasks. In the listening test, Thai numerals from zero to nine were presented auditorily three times each, resulting in a total of 30 trials presented in a randomized order. For each trial, participants were given 5 s to respond. Participants selected the corresponding numeral using a Google Form. If they were unable to respond within the time limit, they were instructed to select “I don't know.”

The arithmetic assessment consisted of 40 addition problems and 40 subtraction problems. In all problems, the operands and the correct answers were restricted to values between zero and nine. A total of 15 min was allotted to complete the arithmetic tasks.

### 2.6 Analysis

#### 2.6.1 Entropy

To quantify the extent to which gaze was distributed across the 24 colors, entropy was computed based on the number of gaze fixations for each color. Specifically, the manufacturer-provided software (Tobii Pro Lab., Tobii AB, Stockholm, Sweden) was used to divide the display into regions corresponding to each of the 24 colors, and the number of fixations within each region was extracted.

Entropy-based measures of gaze behavior have been widely used in studies of gaze dynamics and visual attention [e.g., (Shic et al., 2008; Krejtz et al., 2015; Di Stasi et al., 2016)]. Lower entropy values indicate that gaze was concentrated on a small number of color alternatives (i.e., fewer competing candidates), whereas higher entropy values indicate that gaze was distributed more broadly across multiple color alternatives.

Let *p*_*i*_ denote the proportion of fixations in region (*i*) relative to the total number of fixations. Entropy was calculated as shown in Equation 1:


$$H=-\sum_{i=1}^{24}p_{i}\log_{2}p_{i}$$ (1)

The fixation counts were normalized and scaled to the range [0, 1], and entropy was calculated based on these.

Statistical analyses of entropy were conducted using a linear mixed-effects model. The dependent variable was the entropy value computed separately for each digit (0–9) in each session. Because participants completed repeated tasks across multiple sessions, the resulting observations were not statistically independent. Therefore, a linear mixed-effects model was employed to account for the hierarchical structure of the data and variability between participants. Fixed effects included session (Sessions 1, 2, and 3), stimulus language (Thai numerals or Arabic numerals), and their interaction. To account for individual differences, participant was included as a random intercept in the model. Given the small sample size of the present study, the statistical findings should be interpreted with caution.

In the present study, entropy data were obtained across multiple sessions from each participant, resulting in a hierarchical data structure in which observations were not independent within participants. Accordingly, a linear mixed-effects model was adopted to explicitly model within-subject correlations. Model parameters were estimated using restricted maximum likelihood (REML) (Gueorguieva and Krystal, 2004; Magezi, 2015).

The statistical significance of fixed effects was evaluated using Wald tests. Coefficient estimates, standard errors, *z* values, and exact *p* values are reported. The significance level was set at α = 0.05.

#### 2.6.2 Consistency

Temporal consistency was calculated for each digit from zero to nine by comparing color selections across successive laboratory sessions (i.e., between the first and second sessions, and between the second and third sessions). A score of one was assigned when the same color was selected across sessions, and zero otherwise. Theoretically, consistency scores ranged from zero to a maximum of 10. Consistency was computed separately for Thai numerals and Arabic numerals.

To examine temporal consistency, a two-way repeated-measures analysis of variance (ANOVA) was conducted with interval (between the first and second sessions vs. between the second and third sessions) and stimulus language (Thai numerals vs. Arabic numerals) as within-subject factors. Degrees of freedom and exact *p* values are reported. When the assumption of sphericity was violated, Greenhouse–Geisser corrections were applied. The significance level was set at α = 0.05.

#### 2.6.3 Verbal reports

As described above, participants were encouraged to verbalize their thoughts during the color-selection task, and their utterances were audio-recorded. The audio recordings were transcribed verbatim by a third-party service (Tokyo Hanyaku Co., Ltd., Tokyo, Japan). The resulting transcripts were then used to identify the factors underlying participants' color selections. The coding procedure was conducted independently by three volunteers who were neither experimenters nor participants in the study. Coders were instructed to classify the reported basis for each color selection into one of four categories: shape, meaning, both shape and meaning, or other. A category was assigned to a response when at least two of the three coders agreed on the classification. Participants' verbal reports were translated and reported without modification of their original meaning. Inter-rater reliability was assessed using Fleiss' kappa, indicating substantial agreement among coders (κ = 0.669). Detailed agreement statistics are provided in Supplementary Table S2.

## 3 Results

### 3.1 Dynamics of temporal consistency

**Figure 2:**
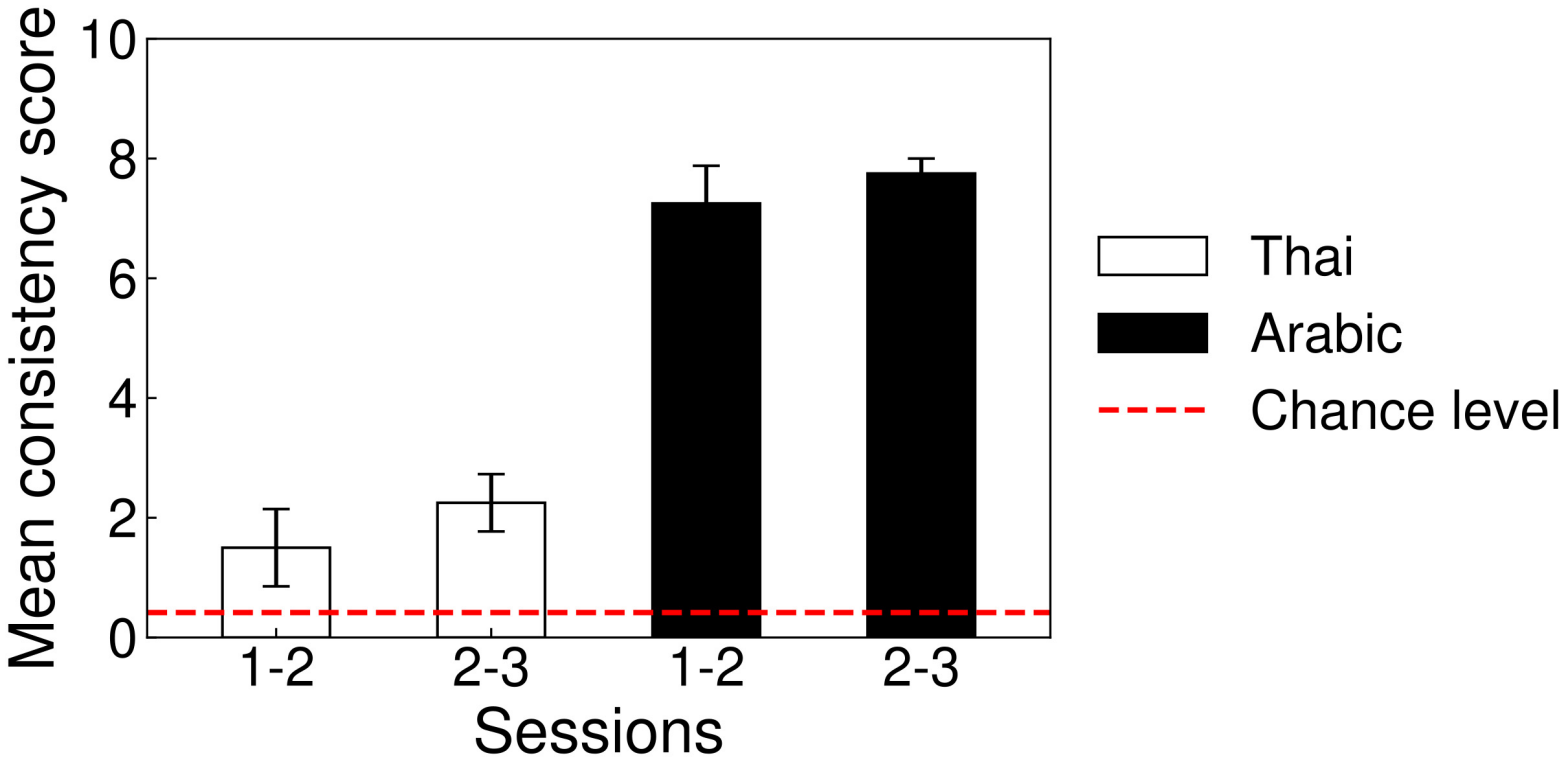
Mean consistency scores (from zero to 10) across participants.

Figure 2 illustrates temporal consistency across laboratory sessions (Session 1–2 and Session 2–3). Error bars represent the standard error of the mean (*s*.*e*.*m*.), and the red dashed line indicates the chance level. The chance level was calculated based on the probability of selecting the same color twice from a palette consisting of 24 colors (1/24). Therefore, the expected consistency score across 10 digits was 0.42 (1/24 × 10 = 0.42). For Arabic numerals, temporal consistency was significantly higher than chance level in both Session 1–2 and Session 2–3 comparisons [one-sample *t*-tests, α = 0.05; Session 1–2: *t*_(3)_ = 10.86, *p* = 0.0017; Session 2–3: *t*_(3)_ = 29.33, *p* < 0.001]. In contrast, for Thai numerals, temporal consistency did not differ significantly from chance level between Session 1 and Session 2 [*t*_(3)_ = 1.68, *p* = 0.19], but became significantly higher than chance level between Session 2 and Session 3 [*t*_(3)_ = 3.83, *p* = 0.031]. In a previous study, non-screened children were asked to select colors for the digits 0–9 from a palette of 13 colors, and their consistency scores did not differ significantly from chance level (Matsuda et al., 2018). In contrast, the participants in the present study exhibited substantially higher levels of temporal consistency than would be expected by chance. These findings suggest that the present participants showed stronger synesthesia-like tendencies than those observed in the general population and provide additional support for their classification as grapheme–color synesthetes.

Taking the consistency score as the dependent variable, a two-way repeated-measures ANOVA was conducted with interval (Session 1–2 vs. Session 2–3) and stimulus language (Thai numerals vs. Arabic numerals) as within-subject factors. No significant main effect of interval was observed [α = 5%, *F*_(1, 3)_ = 9.0, *p* = 0.058]. In contrast, a significant main effect of stimulus language was found [*F*_(1, 3)_ = 360.0, *p* = 0.00032], indicating that overall temporal consistency was lower for Thai numerals than for Arabic numerals. The interaction between interval and stimulus language was not significant [*F*_(1, 3)_ = 1.0, *p* = 0.39]. Because the interaction effect was not significant, no post hoc analyses were conducted. Nevertheless, inspection of the mean values suggests a numerical increase in temporal consistency for Thai numerals from Session 1–2 to Session 2–3.

### 3.2 Entropy of gaze counts

**Figure 3:**
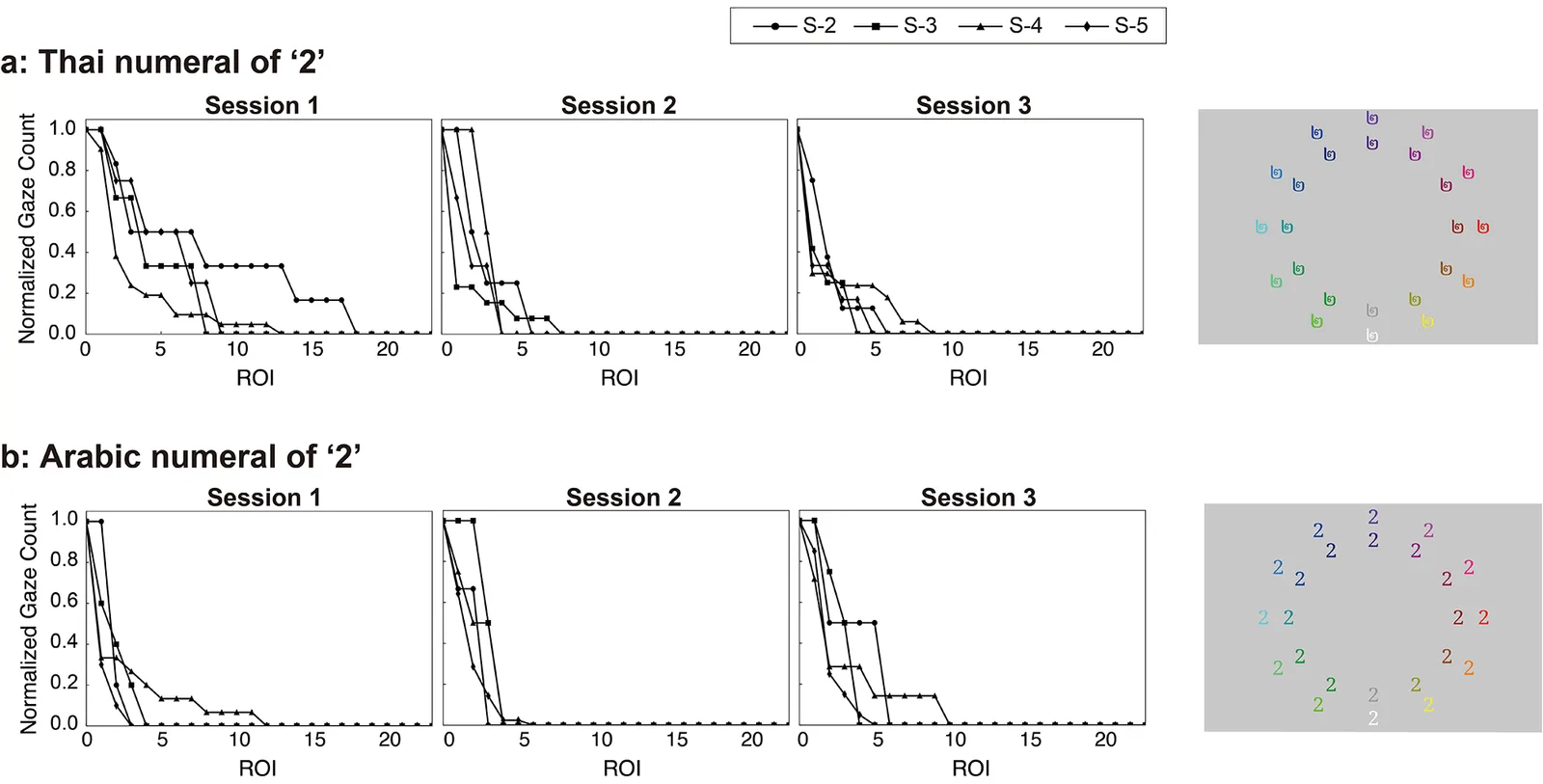
Normalized gaze counts across color regions for the numeral “2” in Thai and Arabic scripts. The image on the far right shows an example of the display at that time. **(a)** Thai numeral “2” across sessions 1–3. **(b)** Arabic numeral “2” across sessions 1–3. Color regions are rank-ordered by fixation frequency, with higher fixation proportions shown on the left.

Figure 3 illustrates, as an example, histograms of gaze counts across color regions for the numeral “2.” The image on the far right shows an example of the display at that time. Along the x-axis, color regions were sorted by gaze count, with the most frequently fixated color region positioned on the left and the least frequently fixated region on the right. The *y*-axis represents the normalized gaze count, calculated as the number of fixations on each color region divided by the total number of fixations for the numeral “2.”

Figure 3a shows the results for Thai numerals, whereas Figure 3b shows the results for Arabic numerals. For Thai numerals (Figure 3a), Session 1 exhibited a broad distribution, indicating that many colors were sampled. In Session 2, the distribution became narrower, and in Session 3, a pronounced peak emerged, indicating strong concentration on a single color candidate. In contrast, for Arabic numerals (Figure 3b), gaze was consistently concentrated on a small number of colors across all sessions. This qualitative pattern suggests that uncertainty in color selection for Thai numerals decreased across sessions, whereas color selection for Arabic numerals remained stable throughout the sessions.

**Figure 4:**
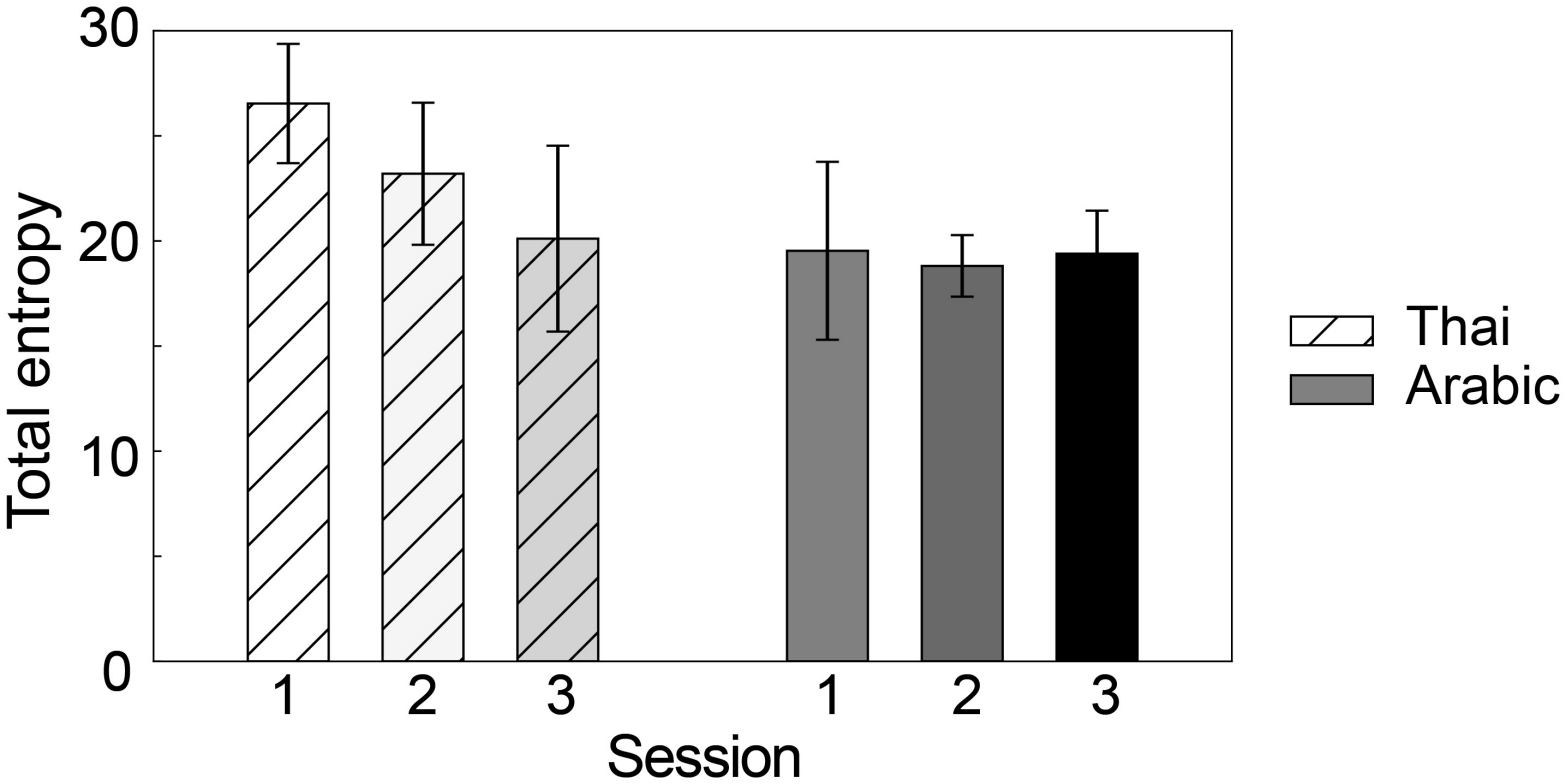
Participant-averaged total entropy values for the digits zero to nine of the normalized gaze counts across the 24 ROIs by each session. The error bars indicate *s*.*e*.*m*.

Figure 4 shows the participant-averaged total entropy values for the digits zero to nine of the normalized gaze counts across the 24 ROIs. Error bars represent the standard error of the mean (*s*.*e*.*m*.). As shown in Figure 4, Thai numerals exhibited higher entropy than Arabic numerals in Session 1; however, entropy for Thai numerals decreased monotonically from Session 1 to Session 3, approaching the level observed for Arabic numerals.

To examine the effects of session and stimulus language on entropy values, a linear mixed-effects model with participants as random intercepts was applied. The model included session (Sessions 1–3), stimulus language (Thai, Arabic), and their interaction as fixed effects. The estimated effect of language was positive [β = 1.03, 95% CI (0.63, 1.44), *p* < 0.001], indicating that entropy values were generally higher for Thai numerals than for Arabic numerals. In contrast, the estimated effect of session was close to zero [β = −0.01, 95% CI (−0.14, 0.13), *p* = 0.918], suggesting little overall change in entropy across sessions when language was not considered.

**Figure 5:**
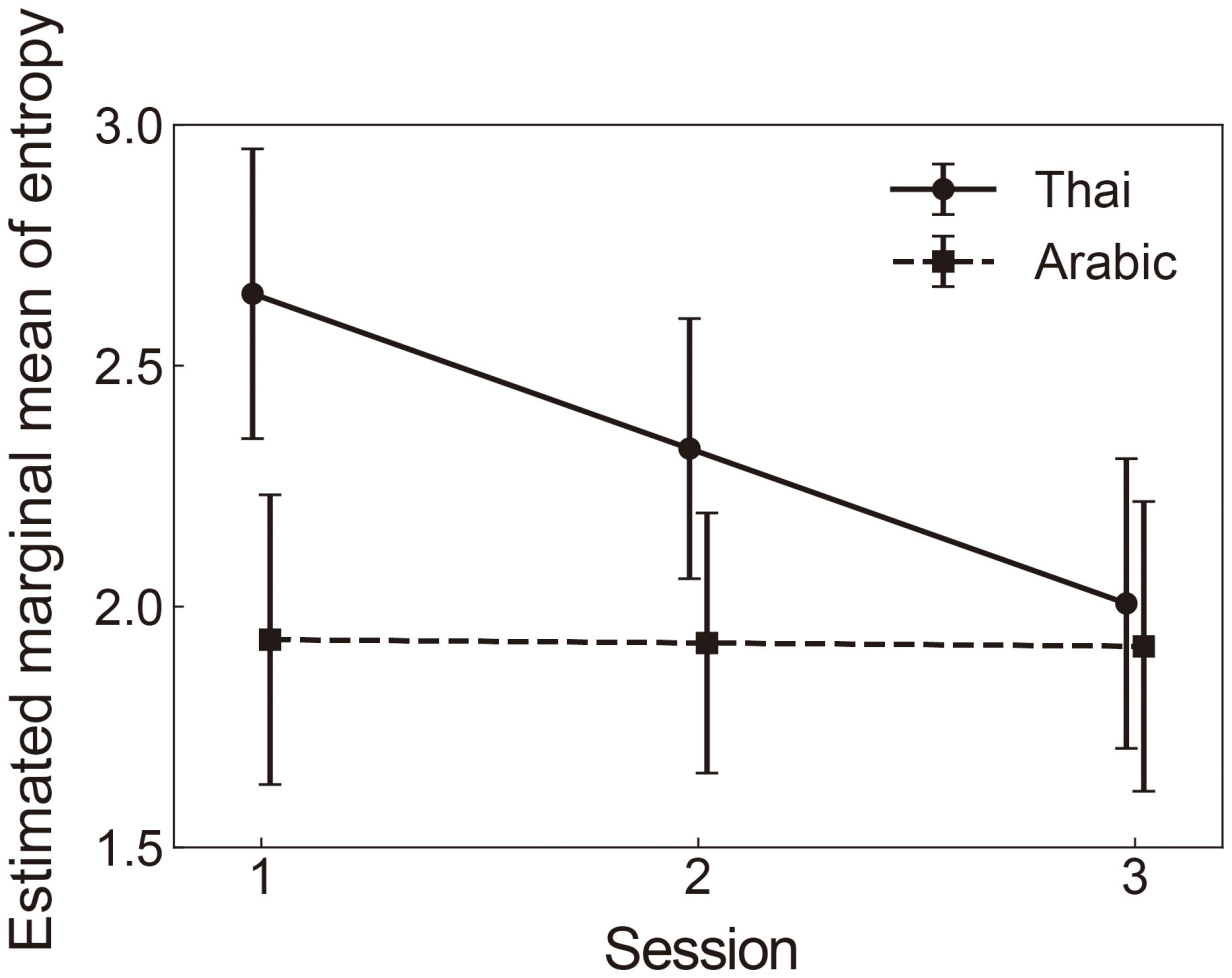
Estimated marginal means of entropy derived from the linear mixed-effects model.

The interaction between session and stimulus language was estimated to be negative [β = −0.31, 95% CI (−0.50, −0.13), *p* = 0.001], indicating that the trajectory of entropy differed between Thai and Arabic numerals. Specifically, entropy values for Thai numerals tended to decrease across sessions, whereas entropy values for Arabic numerals remained relatively stable. This pattern was also reflected in the estimated marginal means shown in Figure 5. The estimated fixed and random effects of the linear mixed-effects model are summarized in Table 2.

**Table 2:** Results of the linear mixed-effects model predicting entropy.

Fixed effect	β	SE	*z*	*p*-Value	95% CI
Intercept	1.94	0.19	10.04	< 0.001	[1.56, 2.32]
Language (Thai)	1.03	0.21	5.00	< 0.001	[0.63, 1.44]
Session	−0.01	0.07	−0.10	0.918	[−0.14, 0.13]
Session × language (Thai)	−0.31	0.10	−3.29	0.001	[−0.50, −0.13]
Random intercept (participant) variance	0.06	0.10			

Session (1–3), stimulus language (Thai vs. Arabic), and their interaction were included as fixed effects, with participant as a random intercept.

To provide a visual summary of these results, the estimated marginal means of entropy derived from the linear mixed-effects model are shown in Figure 5. Figure 5 depicts the model-predicted entropy values for each session while controlling for between-participant variability. Consistent with the statistical results, entropy for Thai stimuli decreased across sessions and approached the level observed for Arabic numerals, whereas entropy for Arabic stimuli remained relatively consistent. This visual pattern aligns with the estimated session-by-language interaction identified in the mixed-effects analysis.

### 3.3 Mapping changes in verbal reports

To probe which cues participants relied on while selecting synesthetic colors for newly learned graphemes, we examined the transcribed verbal reports collected during the eye-tracking task. Each utterance was descriptively coded for the primary cue invoked: Shape (visual form/letterform resemblance), Meaning (digit identity or cross-script correspondence), Shape+Meaning (both), or Unclear (no explicit cue stated; e.g., a color impression without a stated basis). Representative excerpts are provided in Supplementary Table S3.

At Session 1, participants predominantly explained Thai-numeral color choices by referring to visual resemblance (Shape), for example, “looks like M” or “looks like the moon/leaf.” By Session 2, mixed patterns emerged: some utterances continued to rely on Shape, whereas others referenced digit identity (Meaning), such as “when I think it's 9, it almost turns into 9's color.” Session 2 also included meta-comments indicating a fallback to Shape when identity knowledge was not yet accessible [e.g., “I can't remember the meaning of the number, so it ends up being (a color) based on shape”]. By Session 3, utterances more often referenced Meaning (e.g., “like the color of 6/7/8”), and some explicitly blended cues (e.g., “a mix of the color for 3 and the color for M”). In addition, some participants reported vivid color impressions without articulating an explicit cue (coded as Unclear).

## 4 Discussion

This study examined how grapheme–color associations emerge when synesthetes learn a novel grapheme set. A hallmark of grapheme–color synesthesia is the highly reproducible nature of inducer–concurrent pairings over time; thus, we first assessed whether and how temporal consistency changes while a previously unfamiliar script is being acquired. Across three laboratory sessions, temporal consistency for Thai numerals showed a numerical increase (Figure 2), suggesting that the Thai numeral–color mappings gradually stabilized with learning. However, even by Session 3, the consistency for Thai numerals did not reach the level observed for the well-learned script (Arabic numerals), which remained stably high across sessions (Figure 2). This separation implies that our observation window captured an early-to-intermediate stage of consolidation rather than a fully converged endpoint.

To characterize the formation process beyond cross-session agreement in the final selected color, we introduced a gaze-based process measure during the color-selection phase. Specifically, gaze entropy decreased across sessions for Thai numerals relative to Arabic numerals (Figures 4, 5), consistent with a progressive reduction of uncertainty (or competition) among candidate colors as correspondences converged. Finally, qualitative reports aligned with these quantitative trends: participants' explanations suggested a shift in the dominant cues guiding color choices, from reliance on visual form in the earliest session to greater reliance on digit meaning and cross-script identity in later sessions (Supplementary Table S3). Taken together, these converging signatures suggest that emerging mappings are initially supported by readily available form-based cues and become increasingly constrained by identity/semantic information as grapheme knowledge accrues. Below, we elaborate on the implications of gaze entropy as a window onto transitional mapping dynamics and discuss how these dynamics relate to candidate determinants proposed in developmental accounts.

### 4.1 Gaze entropy as a window onto implicit choice dynamics

We computed Shannon entropy from the distribution of fixations across the 24 color options during the selection display. Conceptually, this measure indexes how much pre-decisional competition remains among candidates: higher entropy indicates broadly distributed sampling across many options (extensive exploration), whereas lower entropy indicates concentrated sampling on a smaller subset (a more decisive preference profile). In our mixed-effects analysis, the session-wise change in entropy differed by stimulus language, such that entropy decreased across sessions for Thai numerals but remained relatively stable for Arabic numerals (Figures 4, 5). Because Arabic numerals provided an internal benchmark of mature, well-consolidated mappings, this interaction pattern supports the interpretation that the entropy reduction primarily reflects learning-related convergence for the newly acquired script rather than a global reduction in exploration due to task familiarity.

Importantly, gaze entropy complements temporal consistency because the two measures are sensitive to different stages of acquisition. Temporal consistency is a strict, cross-session criterion (exact agreement in the chosen color), and therefore tends to become informative mainly after mappings have largely stabilized. By contrast, entropy is a within-session, continuous index of how strongly attention (and thus sampling) is constrained to a subset of candidates. Accordingly, entropy can decrease even when the final chosen color is not yet perfectly stable across sessions—for example, when the “winner” color changes between sessions but competition has already narrowed to a small neighborhood of candidates. In this sense, the monotonic reduction in gaze entropy provides process-level evidence for a competition-to-convergence dynamic that precedes (and may ultimately enable) adult-like temporal consistency. Such process measures may help overcome a limitation of purely declarative, self-report–based tests, which are typically most sensitive only after mappings have largely stabilized (Shic et al., 2008; Krejtz et al., 2015; Di Stasi et al., 2016). This interpretation is consistent with broader eye-tracking research showing that gaze allocation patterns provide sensitive indicators of perceptual uncertainty, preference formation, and color-related perceptual processing (Bortolotti et al., 2025b).

### 4.2 From form-based to meaning-based mapping in the transitional period

Focusing on the transitional period of acquiring a new script, we examined how the cues supporting emerging grapheme–color mappings change with learning. As reported in Section 3.3, we descriptively coded participants' utterances during the color-selection task according to the dominant cue invoked: *Shape* (visual form/letterform resemblance), *Meaning* (digit identity and cross-script correspondence), *Shape+Meaning* (both), and *Other/Unclear* (no explicit cue verbalized; representative excerpts are provided in Supplementary Table S3).

Across sessions, the verbal reports suggested a systematic qualitative shift from form-based to meaning-based mapping. In Session 1, when participants had no prior knowledge of Thai numerals, explanations predominantly relied on *Shape* (e.g., “looks like M/leaf/moon”). This pattern is consistent with reports of shape-driven secondary mappings in grapheme–color synesthesia, where visually similar graphemes tend to elicit similar concurrents (e.g., Mills et al., 2002; Brang et al., 2011; Watson et al., 2012). By Session 2, mixed patterns emerged: some utterances continued to appeal to visual resemblance, whereas others began to reference digit identity (e.g., “when I think it is 9, it turns into 9's color”), and meta-comments suggested a fallback to shape when identity knowledge was not yet accessible. By Session 3, utterances more often invoked cross-script identity (e.g., “like the color of 6/7/8”), and some explicitly blended cues (e.g., “a mix of the color for 3 and the color for M”), suggesting that multiple determinants can be concurrently recruited even as mappings become increasingly constrained. This interpretation is also compatible with studies of crossmodal correspondences showing that learned associations can bias perceptual processing and feature integration. Bortolotti et al. demonstrated that color–taste congruency influences visual binding errors, indicating that associative knowledge can affect how perceptual representations are organized (Bortolotti et al., 2025a). In a similar manner, newly acquired grapheme identities may progressively constrain candidate color associations as conceptual knowledge accumulates.

Importantly, this form-to-meaning shift can be interpreted within a pragmatic *perceptual–conceptual* continuum (Matsuda and Matsuda, 2025). Here, “perceptual” refers to cueing tied to the stimulus' sensory characteristics (e.g., its shape), whereas “conceptual” refers to cueing driven by categorical identification (e.g., digit identity), which is expected to be more invariant to surface features. Grapheme–color synesthesia is often regarded as strongly conceptual in its mature form, insofar as concurrents are typically linked to symbolic identity rather than to low-level visual features. Our data suggest that when a grapheme set is newly introduced and identity representations are still weak, perceptual resemblance provides a readily available scaffold; with accumulating knowledge, mappings become increasingly constrained by conceptual identification and can generalize across scripts via digit identity. This interpretation is consistent with accounts in which multiple determinants (shape, phonology, ordinal relations, environmental regularities) compete early and gradually converge through learning (Asano and Yokosawa, 2013; Witthoft and Winawer, 2013), as well as with developmental observations that consistency strengthens with experience (Simner et al., 2009; Simner, 2013).

Notably, the qualitative cue reweighting dovetails with our process measure: the session-wise reduction in gaze entropy for Thai numerals relative to Arabic numerals (Figures 4, 5) indicates that competition among candidate colors narrows as mappings stabilize. Taken together, the verbal reports and gaze dynamics provide converging evidence that emerging correspondences may initially be supported by perceptual heuristics but become progressively shaped by conceptual identity as grapheme knowledge consolidates. Although our study did not directly manipulate perceptual features (e.g., font/size/rotation) to test invariance, the present framework generates a straightforward prediction: as Thai-numeral mappings consolidate, they should become increasingly robust to such surface variations, mirroring the conceptual character of well-learned inducers.

### 4.3 Methodological considerations and limitations

Several limitations qualify the present conclusions. First, the analyzed sample was small (*n* = 4), and one participant was a laboratory member. The limited sample size partly reflects the rarity of grapheme–color synesthesia and the demanding longitudinal design, which required approximately 20 days of participation across laboratory sessions and at-home training. Although the sample was small, the study collected rich behavioral data, including eye-tracking measures that reflect implicit attentional processes. Consequently, the present findings should be regarded as preliminary process-level evidence describing changes observed in this sample rather than definitive evidence that can be generalized to the broader population. Accordingly, the limited sample size may reduce statistical power and constrain the generalizability of the findings. The inclusion of a laboratory member also raises the possibility of expectation or confirmation biases. To assess the robustness of the findings, we repeated the main analyses after excluding the coauthor participant (Supplementary Table S4; Supplementary Figures S3–S5). The overall pattern of temporal consistency and entropy change across sessions remained consistent with the original analysis, and the session-by-language interaction indicating entropy reduction in the Thai condition remained significant [β = −0.241, *SE* = 0.112, *z* = −2.155, *p* = 0.031, 95% CI (−0.461, −0.022)]. Although the main effect of session was no longer significant (β = 0.032, *p* = 0.683), the results suggest that the observed entropy reduction was unlikely to be driven solely by a single participant.

Second, we relied on self-reported synesthesia without administering a standardized diagnostic battery (Eagleman et al., 2007). This is an important limitation because self-report alone cannot establish synesthetic status with the same rigor as standardized screening procedures. Although the present study assessed both Thai numerals and familiar Arabic numerals using the same repeated color-selection procedure, the Arabic numeral consistency task should not be regarded as a substitute for a formal diagnostic battery. Rather, it provides converging behavioral evidence that the participants showed stable grapheme–color associations for familiar numerals. As reported in Section 3, temporal consistency for Arabic numeral–color associations was significantly above the chance-level baseline and was comparable to levels typically used as behavioral markers of grapheme–color synesthesia. Thus, while these findings support the plausibility of the participant classification, the results should be interpreted as being based on self-reported grapheme–color synesthetes with additional behavioral consistency evidence, rather than on a formally diagnosed synesthete sample. Future studies should complement the present protocol with standardized diagnostic screening and larger samples.

Third, our longitudinal window was relatively short (three laboratory sessions), which likely captured an early-to-intermediate stage of association consolidation rather than a fully converged endpoint. Longer follow-up periods are needed to determine whether gaze entropy continues to change after temporal consistency approaches mature levels, and whether the observed cue-reweighting process persists over time.

Finally, potential order and display confounds remain. Within-session numeral order was fixed (0–9), and stimulus language blocks were not counterbalanced, so practice or fatigue effects cannot be completely ruled out. To evaluate the influence of stimulus order, we conducted an additional mixed-effects analysis using Thai numeral presentation order as a predictor. No significant association was observed between presentation order and entropy (β = 0.007, *p* = 0.733), suggesting that the observed entropy changes were unlikely to be explained by stimulus order alone. Furthermore, color locations on the selection wheel remained fixed across trials, and spatial viewing biases could in principle influence fixation distributions. Inspection of the actually selected colors (Supplementary Figures S6, S7) revealed no obvious tendency for participants to repeatedly choose only a restricted subset of wheel locations. Nevertheless, future studies should randomize or counterbalance these design factors to further reduce potential confounds.

### 4.4 Conclusion

In summary, the present longitudinal study makes the transitional formation of novel grapheme–color mappings observable at multiple levels. A numerical increase in temporal consistency for newly learned Thai numerals (relative to stable, high consistency for Arabic numerals), a session-by-language reduction in gaze entropy, and qualitative shifts from form-based to identity/meaning-based explanations together suggest that emerging mappings undergo a reweighting process during learning. These converging signatures are consistent with developmental accounts in which multiple determinants initially compete and gradually converge as grapheme knowledge accumulates (Asano and Yokosawa, 2013). Methodologically, our results highlight gaze entropy as a compact process measure that can complement traditional consistency tests by capturing convergence dynamics before mappings become fully stabilized. Conceptually, they underscore that synesthetic concurrents can function as sensitive markers of learning-related consolidation and offer testable predictions for how perceptual scaffolds give way to more conceptual constraints as a new script becomes familiar.

## Data Availability

The raw data supporting the conclusions of this article will be made available by the authors, without undue reservation.
