# Supplementary material for: Transient acquisition process of grapheme-color synesthesia on novel graphemes
Source: Front Psychol. 2026 Aug 11;17:1821750. doi: 10.3389/fpsyg.2026.1821750 (PMC13503157; doi:10.3389/fpsyg.2026.1821750)
Supplement: Supplementary file 1 [file Data_Sheet_1.pdf]

Supplementary Material

1 TABLES

Table S1. Color stimuli used in the experiment and corresponding RGB values.

Color name	RGB value	Color patch
white	(255, 255, 255)	
gray	(153, 153, 153)	
yellow1	(255, 255, 0)	
yellow2	(153, 153, 0)	
orange1	(255, 128, 0)	
orange2	(153, 76, 0)	
red1	(255, 0, 0)	
red2	(153, 0, 0)	
pink1	(255, 0, 128)	
pink2	(153, 0, 76)	
pink purple1	(255, 0, 255)	
pink purple2	(153, 0, 153)	
purple1	(128, 0, 255)	
purple2	(76, 0, 153)	
dark blue1	(0, 0, 255)	
dark blue2	(0, 0, 153)	
blue1	(0, 128, 255)	
blue2	(0, 76, 153)	
light blue1	(0, 255, 255)	
light blue2	(0, 153, 153)	
light green1	(0, 255, 128)	
light green2	(0, 153, 76)	
green1	(0, 255, 0)	
green2	(0, 153, 0)	

Table S2. Inter-rater reliability statistics for the coding of verbal reports. Inter-rater reliability was assessed using Fleiss' kappa. Complete agreement among all three coders was observed in 66.7% of cases, and agreement among at least two coders was observed in 100.0% of cases.

Measure	Value
Fleiss' κ	0.669
Complete agreement (3/3 coders)	66.7%
Majority agreement ($\geq$2/3 coders)	100.0%

Table S3. Representative transcriptions of verbal reports during the color-selection phase of the eye-tracking task. Utterances were transcribed verbatim and anonymized. Primary explanatory cues were descriptively coded as Shape, Meaning, or Shape+Meaning.

Session	Participant	Arabic	Thai	Verbal Description	Cue
1	S-3	3	๓	It looks like “M”, so brown.	Shape
		4	๔	It looks like the moon, so yellow.	Shape
	S-4	3	๓	Maybe I remember it as magenta because it looks like “M”.	Shape
		4	๔	It also looks like a leaf, so a natural and breezy feeling—light green.	Shape
		6	๖	It resembles a “C”, so yellow.	Shape
		9	๙	It looks like a coconut with fruit, so maybe brown.	Shape
2	S-2	4	๔	I have an image of it being a pale and dark color. Maybe a dark orange.	–
	S-3	4	๔	I can’t remember the meaning of the number, so it ends up being yellow based on shape.	Shape
		5	๕	When I think it’s 9, it almost turns into 9’s color.	Meaning
		6	๖	Based on the shape, I think it’s white.	Shape
3	S-3	3	๓	It’s a mix of the color for 3 and the color for M—like a mild brown and pink.	Shape+Meaning
		6	๖	Definitely brown, like the color of 6.	Meaning
		7	๗	Similar to the color of 7.	Meaning
		8	๘	It’s close to 8’s color, but a bit darker.	Meaning
	S-5	0	๐	I always see a deep red tone very clearly.	–

Table S4. Results of the linear mixed-effects model predicting entropy using data excluding the laboratory-member participant to assess the robustness of the findings against potential expectation or confirmation bias. Session (1–3), stimulus language (Thai vs. Arabic), and their interaction were included as fixed effects, with participant as a random intercept.

Fixed effect	β	SE	z	p	95% CI
Intercept	1.85	0.25	7.45	$< .001$	[1.36, 2.34]
Language (Thai)	0.94	0.24	3.88	$< .001$	[0.47, 1.41]
Session	−0.03	0.08	0.41	.68	[−0.12, 0.19]
Session × Language (Thai)	−0.24	0.11	−2.16	.031	[−0.46, −0.02]
Random intercept (Participant) variance	0.10	0.17			

2 FIGURES

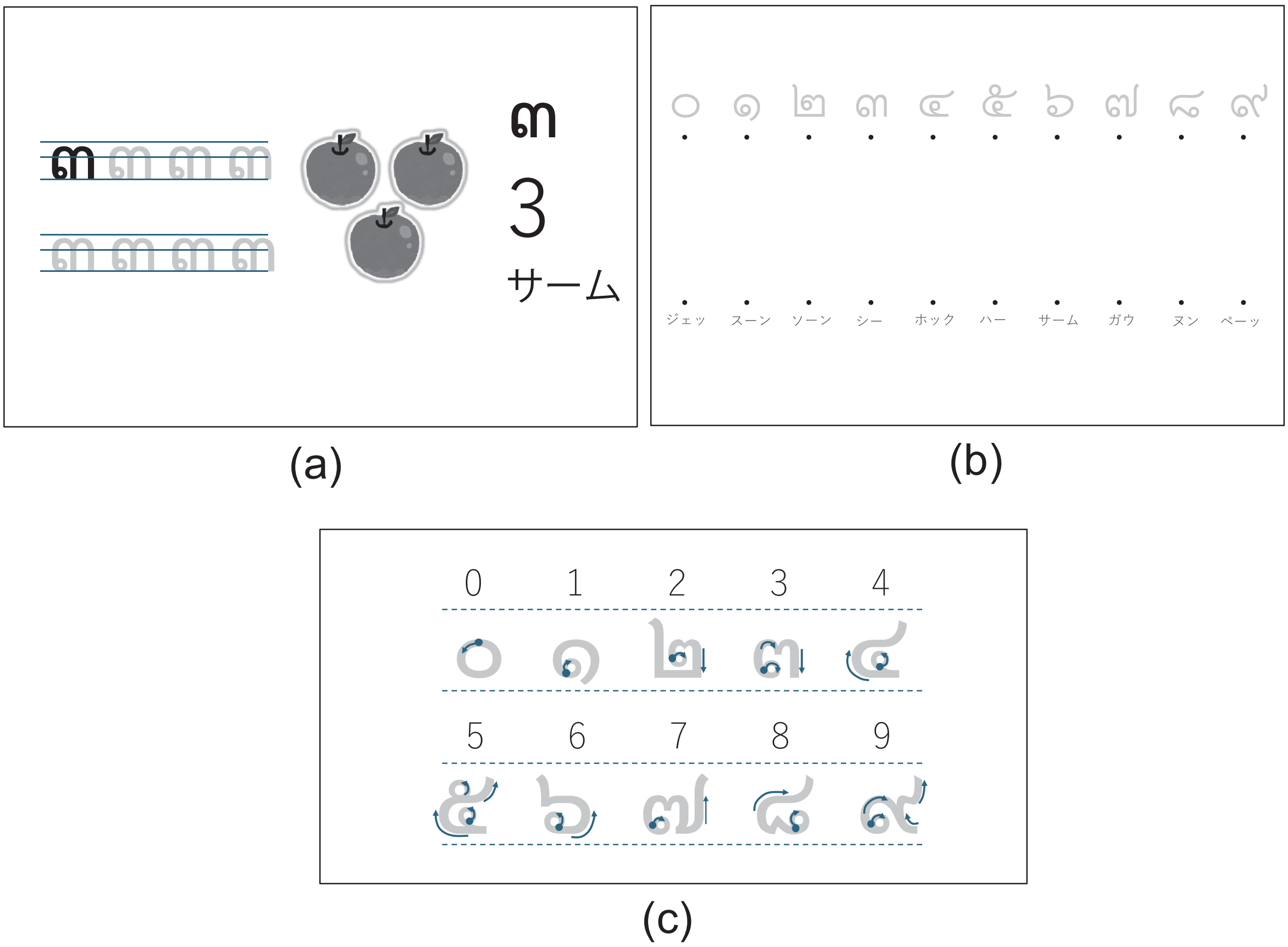

Figure S1. Learning materials used during the initial home-training phase (Days two to eight). The single composite image includes three components: (a) practice of a specific Thai numeral, (b) mapping between Thai numerals and their pronunciations, and (c) tracing exercises for Thai numerals from zero to nine.

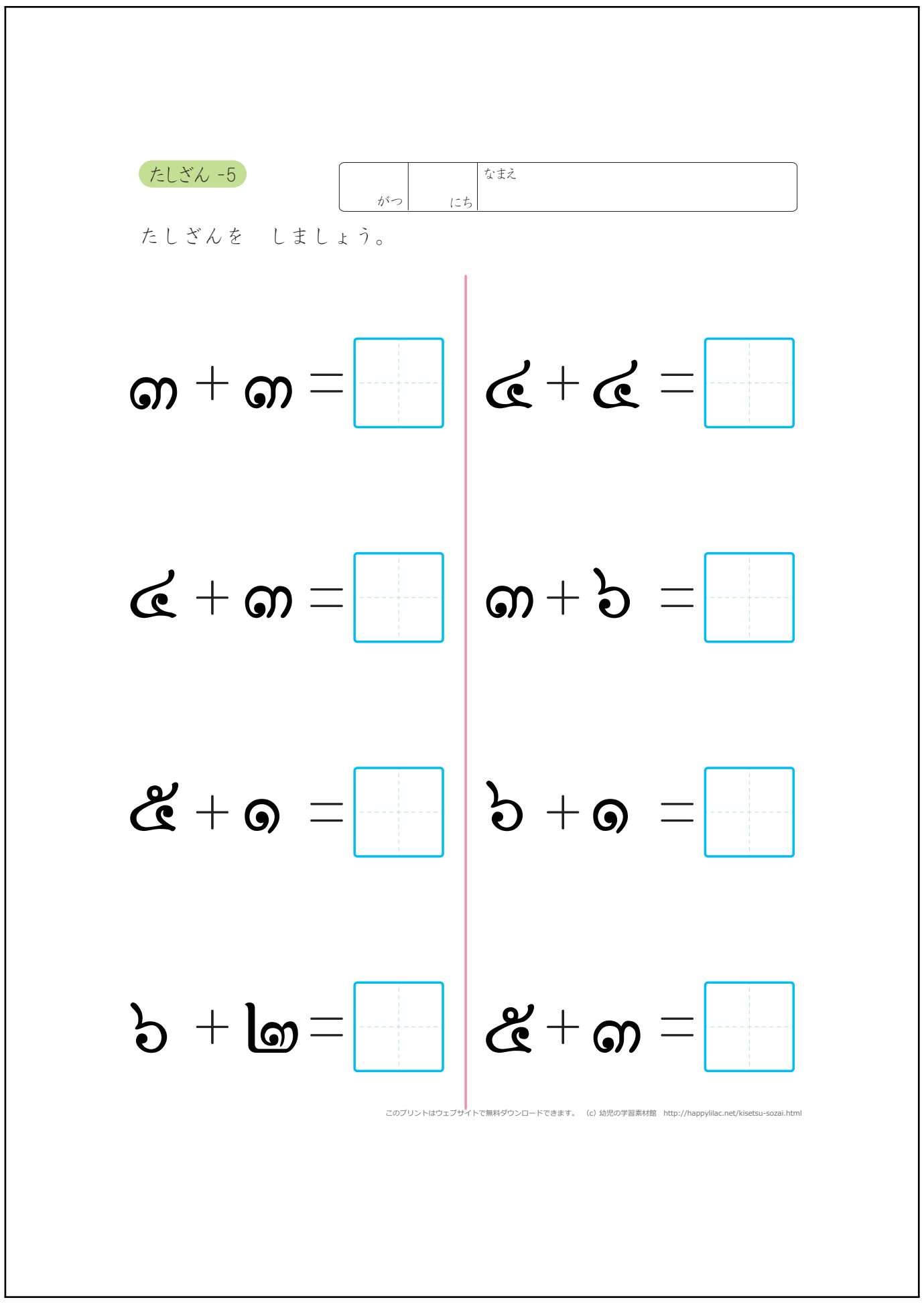

Figure S2. Learning materials used during the subsequent home-training phase (Days 10–19). An example of the addition exercises is presented.

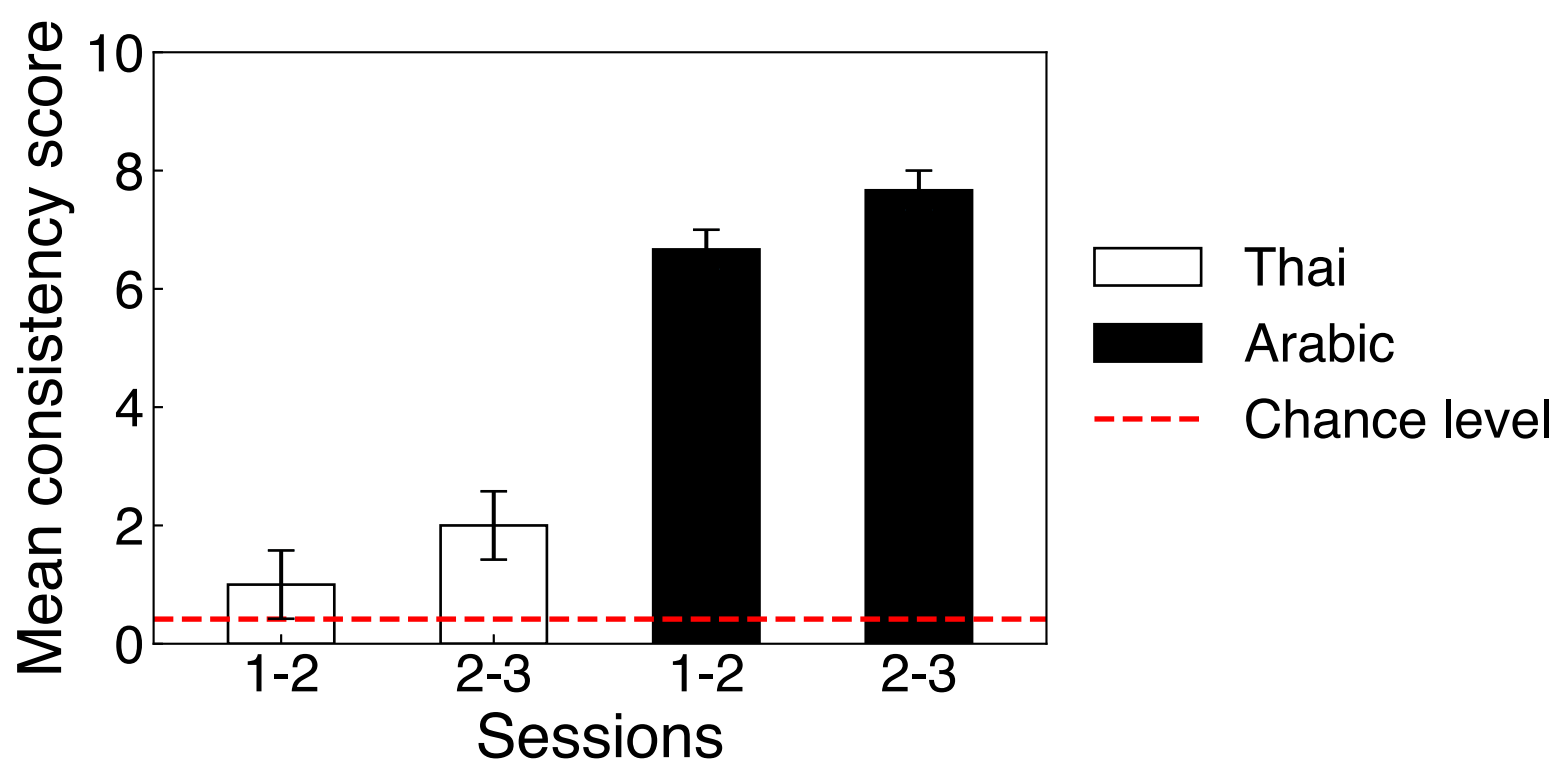

Figure S3. Mean consistency scores (from zero to 10) using data excluding the laboratory-member participant to assess the robustness of the findings against potential expectation or confirmation bias..

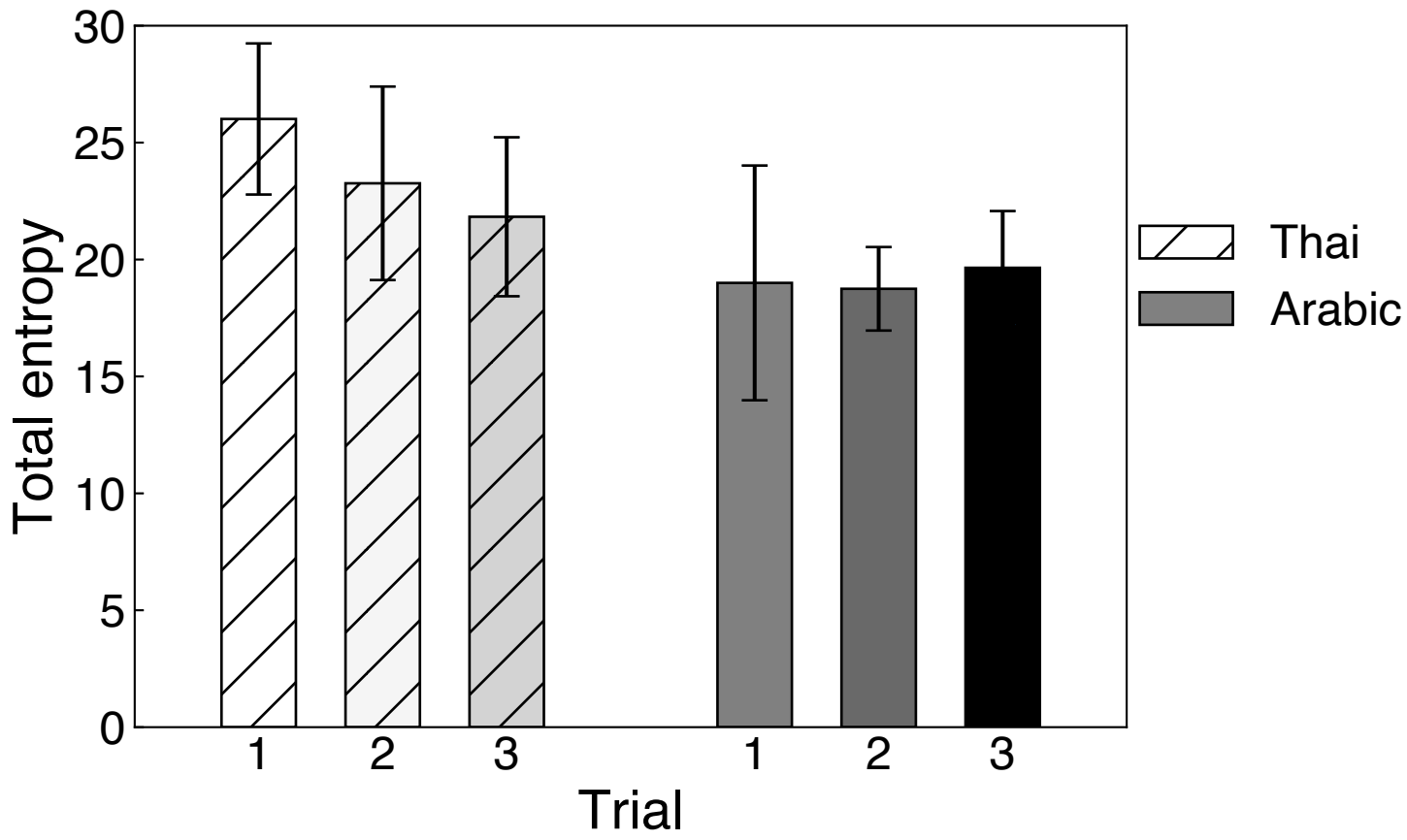

Figure S4. Participant-averaged total entropy values for the digits zero to nine of the normalized gaze counts across the 24 ROIs by each Session. This figure uses data excluding the laboratory-member participant to assess the robustness of the findings against potential expectation or confirmation bias. The error bars indicate $s.e.m.$

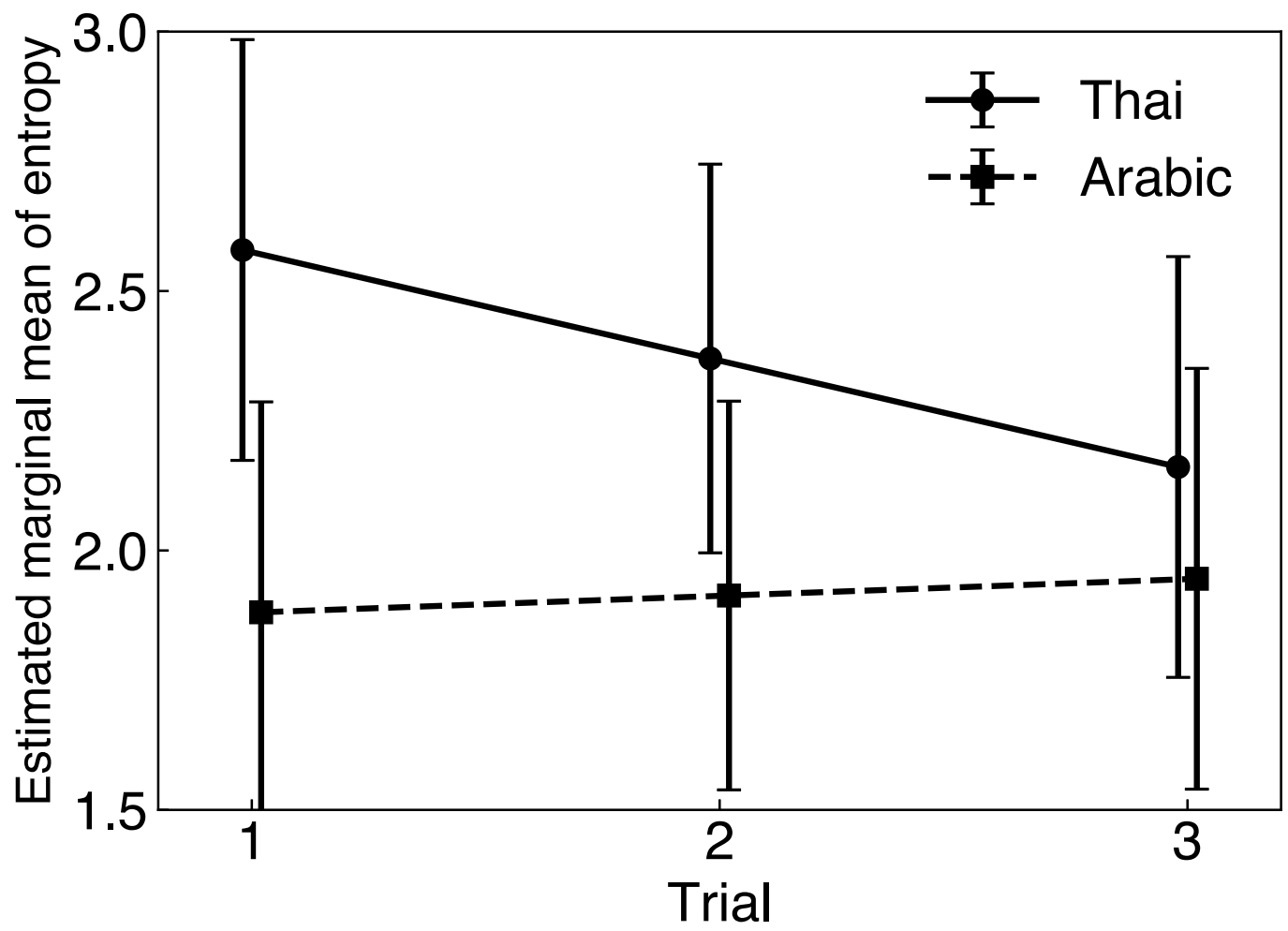

Figure S5. Estimated marginal means of entropy derived from the linear mixed-effects model.This figure uses data excluding the laboratory-member participant to assess the robustness of the findings against potential expectation or confirmation bias.

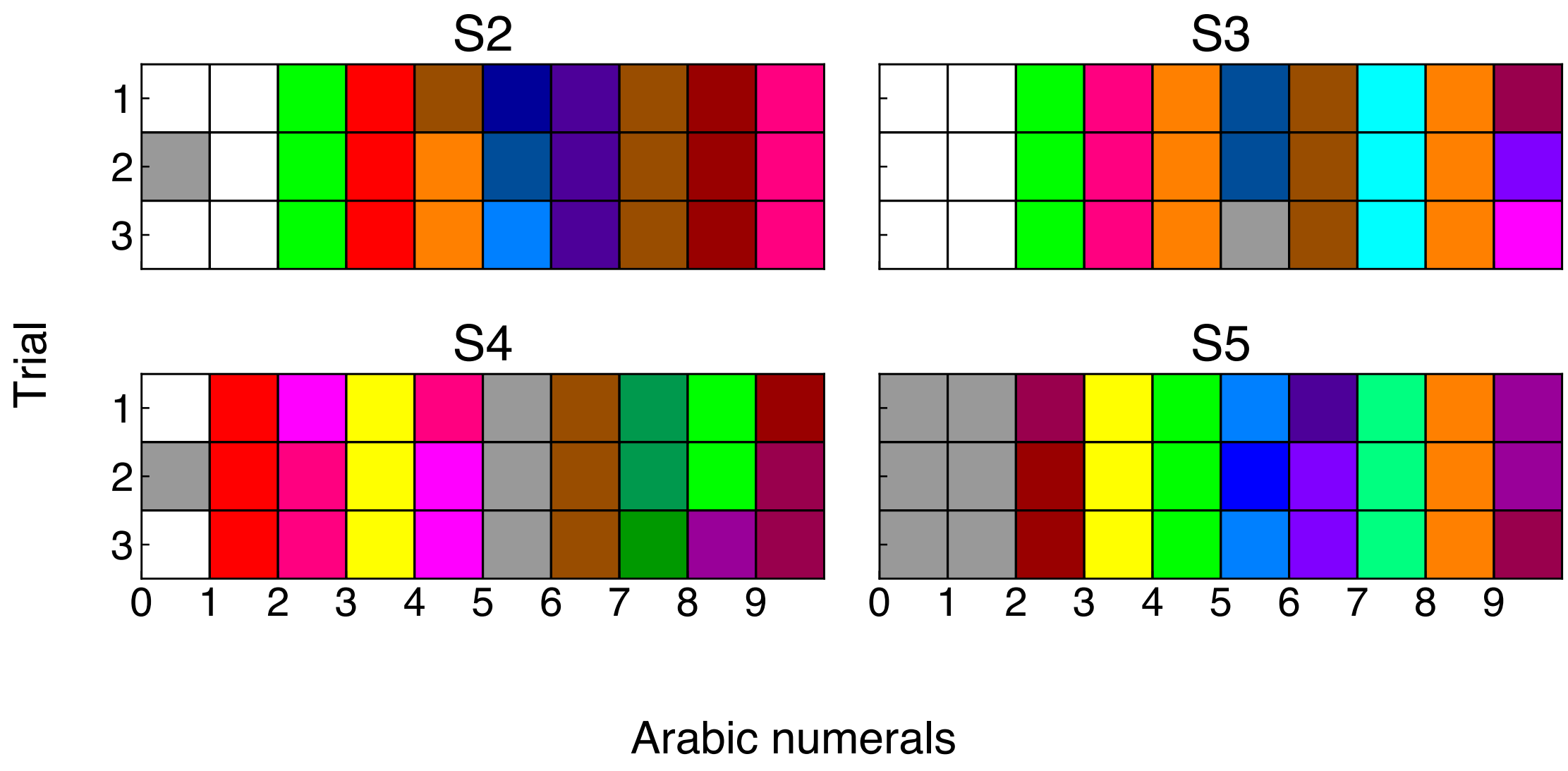

Figure S6. Colors selected for Arabic numerals (0–9) by each participant across the three experimental sessions.

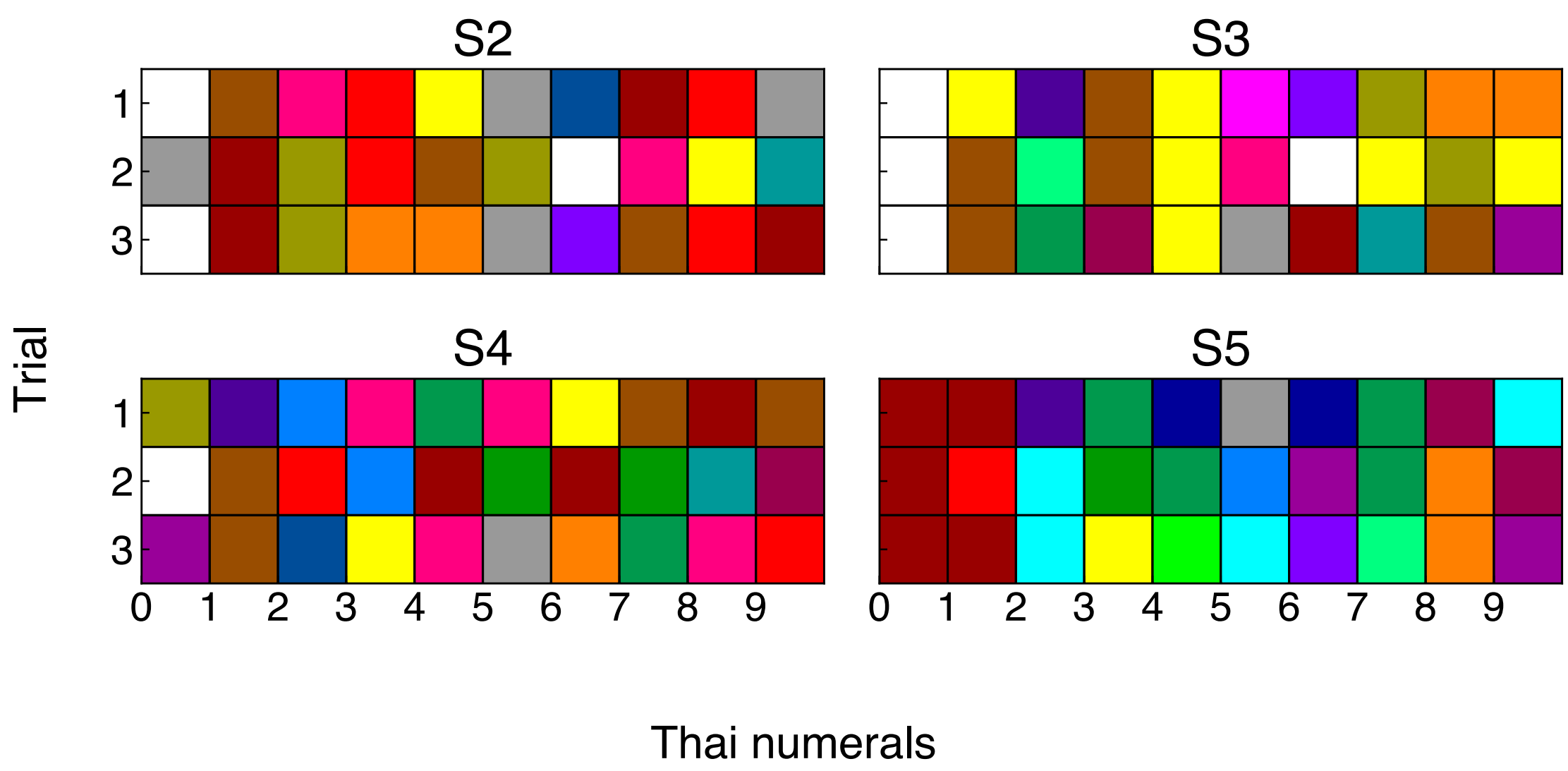

Figure S7. Colors selected for Thai numerals (0–9) by each participant across the three experimental sessions.
